# Supplementary figures and images for: Plant-exuded chemical signals induce surface attachment of the bacterial pathogen Pseudomonas syringae
Source: PeerJ. 2023 Mar 27;11:e14862. doi: 10.7717/peerj.14862 (PMC10062345; doi:10.7717/peerj.14862)

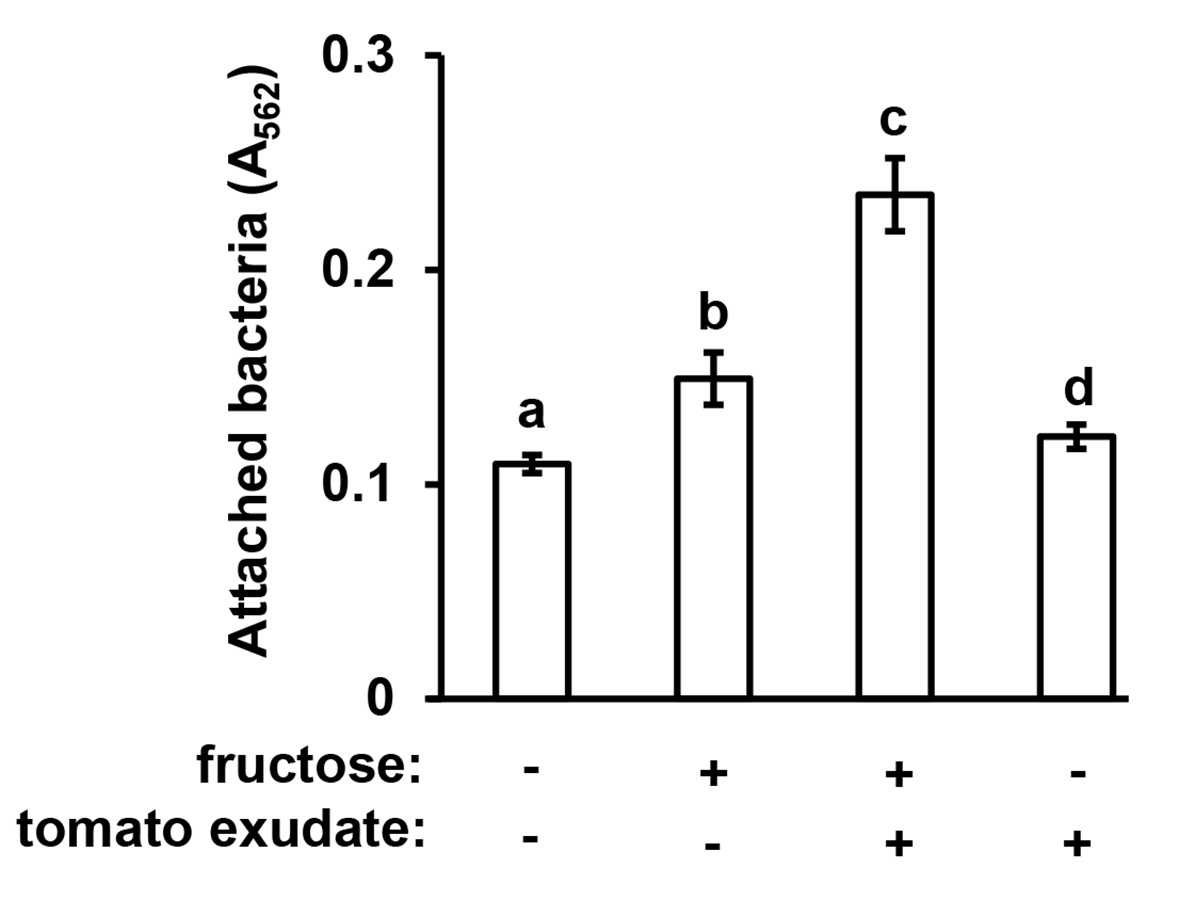

Supplement: Supplemental Information 1 — P. syringae DC3000 were cultured in minimal medium (MM) alone, or MM supplemented with 50 mM fructose and/or tomato leaf exudate. After 16 h, assay plate wells were washed with water to remove unattached bacteria, then stained with crystal violet (CV). Graphed are means of Absorbance (A562) measurements of CV-stained microtiter plate wells. Small case letters denote statistical significance groupings based on pairwise t-tests, p < 0.05. Error bars are standard error; n = 4. Data are representative of three independent experiments. [file peerj-11-14862-s001.png]

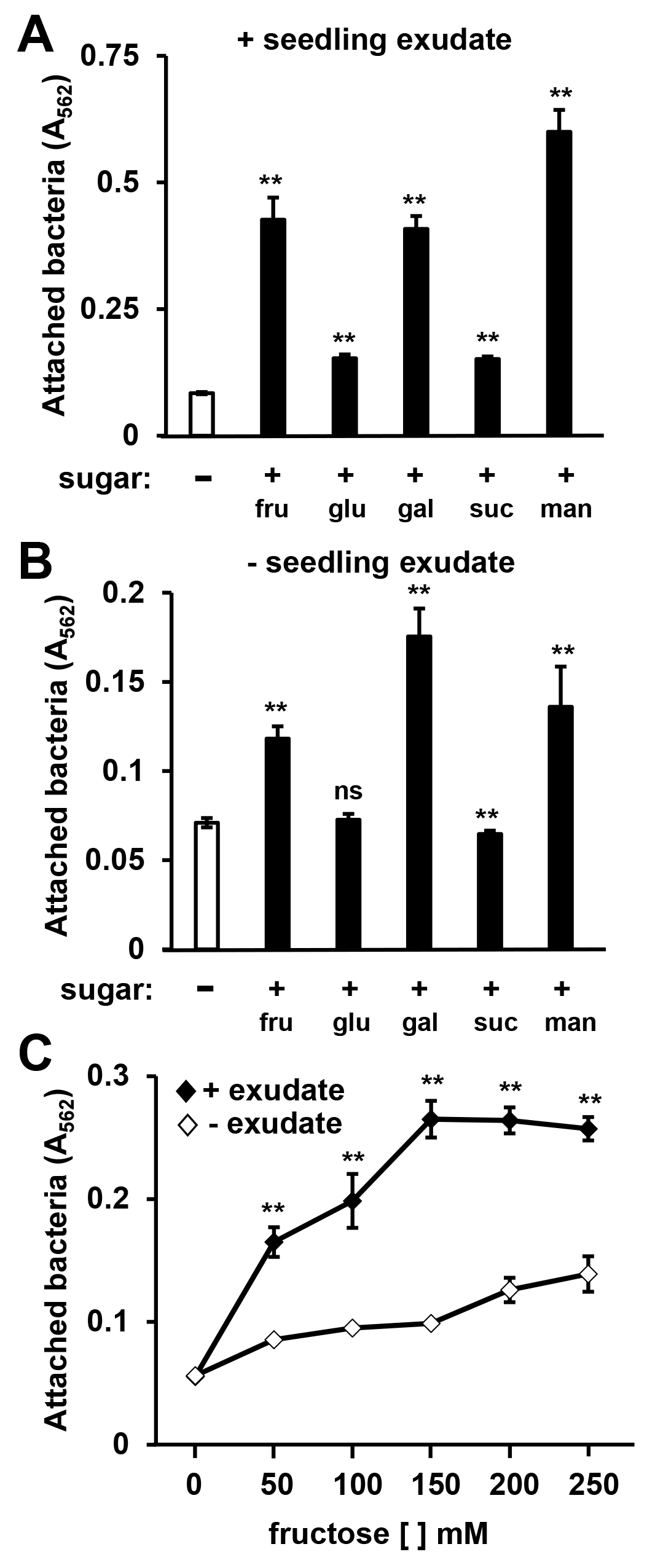

Supplement: Supplemental Information 2 — P. syringae DC3000 were cultured for 16 h in minimal medium (MM) alone or MM supplemented with 50 mM fructose and Arabidopsis seedling exudate. Attached and planktonic bacteria were enumerated by serial dilution plating on KB agar and counting of colony-forming units (cfus). (A) Graphed are means of percent attached cfus vs total cfus (attached plus planktonic). Error bars are standard error; n = 4. (B) Graphed are means of log total cfus (planktonic and attached). Error bars are standard error, n = 4. Asterisks indicate statistical significance based on pairwise t-tests, *** p < 0.001. Data are representative of at least three independent experiments. [file peerj-11-14862-s002.png]

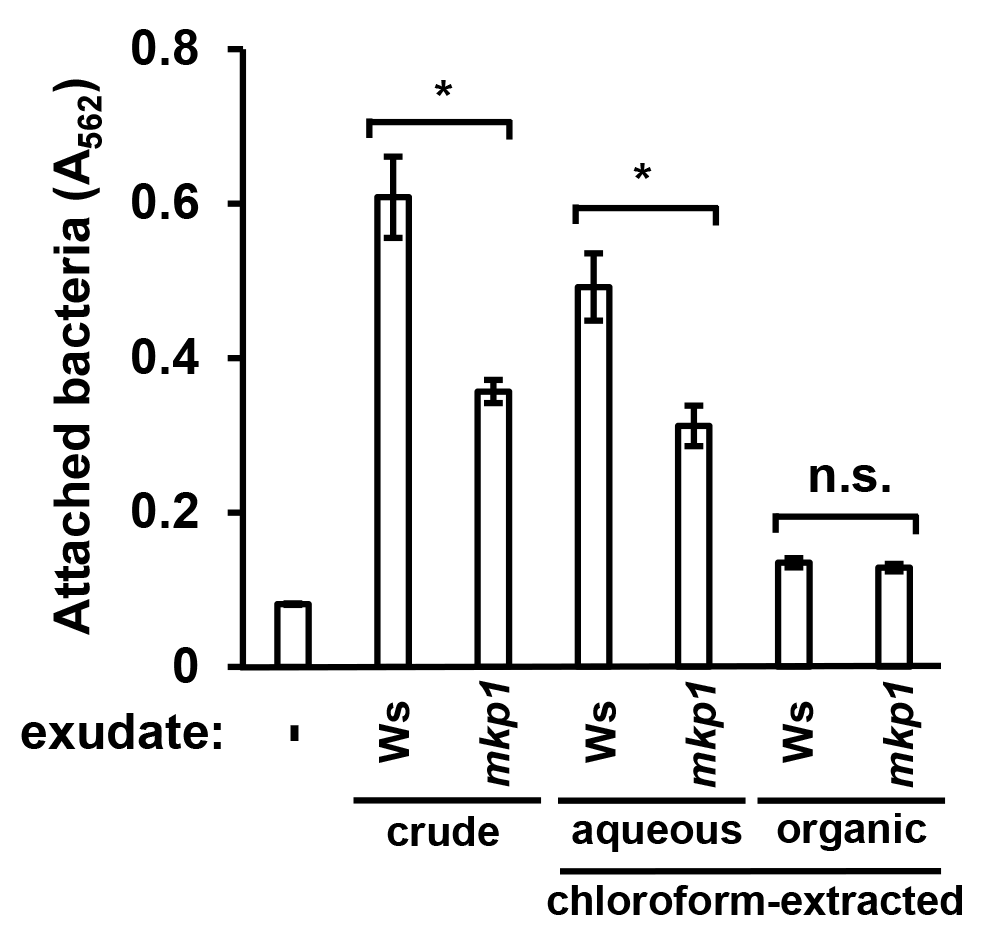

Supplement: Supplemental Information 3 — DC3000 were cultured for 16 h in (A) Arabidopsis seedling exudate or (B) water in either minimal medium (MM) only or MM supplemented with 50 mM fructose (fruc), glucose (gluc), galactose (gal), sucrose (suc), or mannitol (man). Graphed are means of Absorbance (A562) measurements of crystal violet (CV)-stained wells after incubating cultures. Error bars are standard error; n = 6. (C) DC3000 were cultured with or without Arabidopsis seedling exudate in MM supplemented with varying concentrations of fructose. Graphed are means of A562 measurements of CV-stained wells after incubating cultures for 16 h. Error bars are standard error; n = 4. Asterisks indicate statistical significance based on pairwise t-tests, ** is p < 0.01, ns is not significant. [file peerj-11-14862-s003.png]
